# Effect of Cyclic Strain on Cardiomyogenic Differentiation of Rat Bone Marrow Derived Mesenchymal Stem Cells

**DOI:** 10.1371/journal.pone.0034960

**Published:** 2012-04-04

**Authors:** Yan Huang, Lisha Zheng, Xianghui Gong, Xiaoling Jia, Wei Song, Meili Liu, Yubo Fan

**Affiliations:** Key Laboratory for Biomechanics and Mechanobiology of Ministry of Education, School of Biological Science and Medical Engineering, Beihang University, Beijing, China; University of California, Merced, United States of America

## Abstract

Mesenchymal stem cells (MSCs) are a potential source of material for the generation of tissue-engineered cardiac grafts because of their ability to transdifferentiate into cardiomyocytes after chemical treatments or co-culture with cardiomyocytes. Cardiomyocytes in the body are subjected to cyclic strain induced by the rhythmic heart beating. Whether cyclic strain could regulate rat bone marrow derived MSC (rBMSC) differentiation into cardiomyocyte-like lineage was investigated in this study. A stretching device was used to generate the cyclic strain for rBMSCs. Cardiomyogenic differentiation was evaluated using quantitative real-time reverse transcription polymerase chain reaction (RT-PCR), immunocytochemistry and western-blotting. The results demonstrated that appropriate cyclic strain treatment alone could induce cardiomyogenic differentiation of rBMSCs, as confirmed by the expression of cardiomyocyte-related markers at both mRNA and protein levels. Furthermore, rBMSCs exposed to the strain stimulation expressed cardiomyocyte-related markers at a higher level than the shear stimulation. In addition, when rBMSCs were exposed to both strain and 5-azacytidine (5-aza), expression levels of cardiomyocyte-related markers significantly increased to a degree suggestive of a synergistic interaction. These results suggest that cyclic strain is an important mechanical stimulus affecting the cardiomyogenic differentiation of rBMSCs. This provides a new avenue for mechanistic studies of stem cell differentiation and a new approach to obtain more committed differentiated cells.

## Introduction

Recently, mesenchymal stem cells (MSCs) have shown applicability as a cell source for the regeneration of cardiac tissue damaged by myocardial infarction (MI) [1]. MSCs could exhibit cardiomyocyte-like phenotypes following 5-azacytidine (5-aza) treatment [2], [3], addition of growth factors [4], [5], co-culture with normal cardiomyocytes [6], [7], or in conditioned medium obtained from cardiomyocytes after hypoxia/reoxygenation [8]. MSCs transplanted into infarcted myocardium have been shown to regenerate cardiac tissue, thicken the wall of the infarcted regions, and improve cardiac functions [9], [10]. However, in those *in vitro* and *in vivo* experiments, only a very small proportion of MSCs showed a cardiomyocyte phenotype, so their potential clinical benefits for cardiac repair are quite limited [11]. A new and effective method is urgently needed to induce MSC to differentiate into cardiomyocytes.

The biochemical environment has long been known to govern MSC differentiation, but more recent evidences show that the physical environment can also direct the cell functions [12]–[15]. The *in vitro* mechanobiological experiments demonstrated that mechanical loadings could regulate MSC proliferation [16], influence MSC cytoskeleton [17] and affect the osteogenic, chondrogenic or endothelium oriented phenotype of MSCs [16], [18]–[20]. For example, Kreke et al. reported that the application of 2.3 dyne/cm^2^ fluid shear stress (FSS) on MSCs cultured in osteogenic media increased the expression of mRNA for different osteogenic markers [21]–[23]. Our group and other researchers have showed that the combined stimulus of VEGF and fluid shear stimulation of approximately 15 dyne/cm^2^ enhanced MSC differentiation into endothelial cell [24], [25]. We also reported that 10 dyne/cm^2^ FSS could mediate MSC differentiation into cardiomyocyte-like cells [26]. In addition to shear stress, continuous 10% uniaxial straining promoted the expression of genes related to smooth muscle cells (SMCs) when MSCs were cultured on smooth surfaces [27]. Another report has suggested that application of cyclic strain (3%, 0.25 Hz) to MSCs cultured in osteogenic media stimulated a 2.3-fold increase in matrix mineralization over unstrained cells [28]. Recent studies have shown that cyclic strain could promote cardiomyogenic differentiation of embryonic stem cells (ESCs) [29]–[31]. However, the effects of cyclic strain on MSC differentiation into cardiomyocyte are not yet well understood. Cardiomyocytes in the body are subjected to cyclic mechanical strain induced by the rhythmic heart beating [29]. Studies have demonstrated that mechanical stretching could affect cardiomyogenesis [32]. Neonatal or adult cardiomyocytes increased the expression of cardiomyocyte-related factors in response to static or dynamic stretching [33], [34].

In a search for new methods to induce MSC to differentiate into cardiomyocytes for cardiac therapies and to gain a better understanding of the role of mechanical loads in cardiovascular development and remodeling, we investigated the effects of cyclic strain on cardiomyogenic differentiation of MSCs *in vitro* in this study. Rat bone marrow derived MSCs (rBMSCs) were used in this study, which are abundant in source, have small differences in different batches of animals and are easy to be used in the subsequent research on transplantation. A self-designed mechanical stretching device was used in this study, which could generate cyclic strain with different magnitude, frequency or duration on cultured cells, and monitor many parameters of reaction system including temperature, pH, pO_2_ and pCO_2_. We specifically addressed the following questions. (1) Is the cardiomyogenic differentiation of rBMSCs influenced by cyclic strain? (2) Is the cardiomyogenic differentiation of rBMSCs related to the magnitude, duration or frequency of mechanical strain? (3) Which is a more potent stimulant for rBMSC differentiation toward the cardiomyocyte lineage, cyclic strain or FSS? (4) Is there a synergistic or antagonistic effect between the biochemical regents and mechanical treatments for the cardiomyogenic differentiation of rBMSCs?

## Materials and Methods

### Ethics Statement

All experiments involving the use of animals were in compliance with Provisions and General Recommendation of Chinese Experimental Animals Administration Legislation and were approved by Beijing Municipal Science & Technology Commission (Permit Number: SCXK (Beijing) 2006-0008 and SYXK (Beijing) 2006-0025).

### Cell culture and preparation

rBMSCs were isolated from the femurs and tibias of 30-day-old male Sprague-Dawley rats (Peking University Laboratory Animal Center, Beijing, China) as previously described [26]. The experiment of rBMSC isolation was conducted at least seven times. In each experiment, the bone marrow from two rats was combined to increase cell yield. Density gradient centrifugation was performed to isolate cells from bone marrow using the percoll technique (Pharmacia, Uppsala, Sweden). Isolated cells were plated in four 25-cm^2^ flasks and cultured in Dulbecco's modified Eagle medium-low glucose (DMEM-LG; Gibco, Grand Island, NY) supplemented with 10% fetal bovine serum (Gibco) for 3 days at 37°C in a humidified atmosphere containing 5% CO_2_. After 3 days of culture, nonadherent cells were removed, and the remaining cells were then expanded to 90% confluency. About 3×10^6^ cells could be harvested from per flask by 0.25% trypsin/0.01% EDTA (Sigma, St. Louis, MO). rBMSCs in passages 2–4 were used for subsequent experiments. Myocytes were isolated from the hearts of neonatal Sprague-Dawley rats (1 to 3 days old) using the neonatal cardiomyocyte isolation methods [35].

### Mechanical device

**Figure 1:**
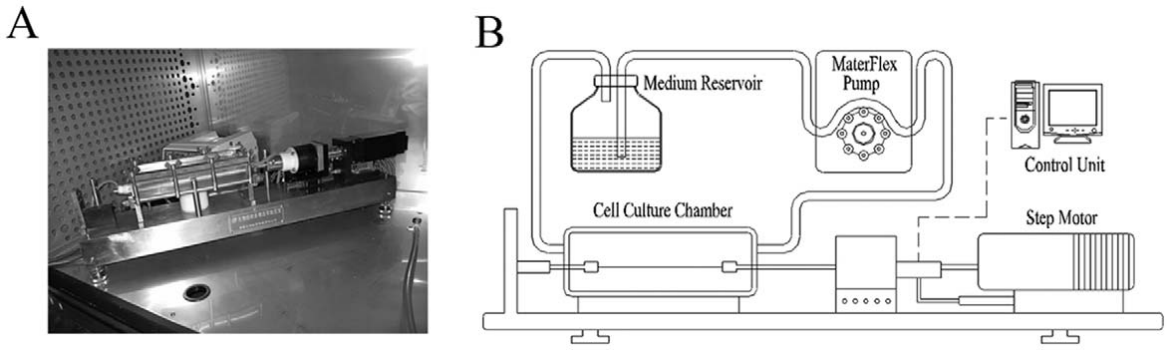
Stretch device. (A) Mechanical stretching device used in our laboratory. (B) Schematic representation of uniaxial stretch device.

A self-designed mechanical stretching device was used to apply cyclic strain to cultured cells, which consists of a tissue culture system and a tensile generator. The tissue culture system consists of a cell culture chamber, a reservoir of culture medium and a MaterFlex pump. The tensile unit contains a step motor, a step motor driver, a signal amplifier and a control unit (PC computer) (Fig. 1). rBMSCs were seeded on an elastic silicone membrane at 3×10^4^ cells/cm^2^ which was mounted to the cell culture chamber. The strain was generated by shrinking and expanding of elastic silicone membrane. The strain applied on the membrane was transferred to the attached rBMSCs. The amplitude, frequency and duration of strain were controlled by the computer. The system was kept at 37°C and equilibrated with 95% humidified air containing 5% CO_2_.

A parallel-plate flow chamber was used to shear cultured rBMSCs as previously described [26]. In brief, rBMSCs were seeded on a glass microscope slide at 3×10^4^ cells/cm^2^. A silicone gasket was sandwiched between the glass slide and an acrylic plate to create a rectangular flow channel. Different magnitudes of shear stress were generated by the flow across the channel resulting from the height difference between two reservoirs. The system was kept at 37°C and equilibrated with 95% humidified air containing 5% CO_2_.

### Quantitative real-time reverse transcription polymerase chain reaction (RT-PCR) analysis

The mRNA levels of GATA4, β-MHC, NKx-2.5, MEF-2c and glyceraldehyde-3-phosphate dehydrogenase (GAPDH) were analyzed by quantitative real-time RT-PCR. Total RNA was extracted from cultured cells using TRIzol reagent (Invitrogen, Carlsbad, CA) according to the manufacturer's protocol, and quantified using a Genequant pro RNA/DNA calculator (Bio-Rad, Hercules, CA). cDNA was prepared from 1 µg total RNA using a cDNA synthesis kit according to manufacturer's instructions (Promega, Madison, WI). The forward and reverse sequences of the primers (synthesized by Invitrogen) in quantitative PCR are listed in Table 1. A 1 µl cDNA sample was added to 5 nmol of each primer, 10 µl of 2×SYBR Green Supermix (Takara, Kyoto, Japan) and PCR-grade water to a volume of 20 µl. Three replicas were performed in the real-time RT-PCR analysis. Real-time PCR was performed in an iCycler iQ real-time PCR detection system (Bio-Rad). Controls were performed with no reverse transcription or water for each gene to demonstrate the specificity of the primers and the lack of DNA contamination in samples. PCR cycling conditions were as follows: initial 95°C for 30 seconds, then 40 cycles using 95°C for 10 seconds, and 58°C for 35 seconds. Melt curve analysis was performed on the iCycler over the range 55°C to 95°C by monitoring iQ SYBR green fluorescence with increasing temperature (0.5°C increment changes at 10-second intervals). Quantification of the results was done using the comparative CT method [36] and for internal normalization, the housekeeping gene GAPDH was employed. The standard curves were generated by serial dilutions of sample cDNA in five 10-fold dilution steps and used for regression analyses. The amplified product (5 µl) was separated on a 2% agarose gel and the bands were visualized by ethidium bromide staining.

**Table 1:** Primers for real-time RT-PCR.

Gene	Forward primer	Reverse primer	Size (base pairs)
GATA4	5′-AGAAGGCAGAGAGTGTGTCA-3′	5′-CAGTGTGGTGGTGGTAGTCT-3′	208
β-MHC	5′-ATCAAGGGAAAGCAGGAAGC-3′	5′-CCTTGTCTACAGGTGCATCA-3′	196
NKx-2.5	5′-ACCCTCGGGCGGATAAGAA-3′	5′-GACAGGTACCGCTGTTGCTTGA-3′	178
MEF-2c	5′-GCAGACGATTCAGTAGGT-3′	5′-CCAGTGGCAGAAGATTAG-3′	211
GAPDH	5′-TGTTCCTACCCCCAATGTATCCG-3′	5′-TGCTTCACCACCTTCTTGATGTCAT-3′	90

### MTT measuring

The proliferation of cultured rBMSCs was determined by measuring the reduction of 3-(4, 5-dimethylthiazol-2-yl)-2, 5-diphenyltetrazolium bromide (MTT) to formazan [37].

### Antibodies and immunostaining

Cells were fixed in 4% paraformaldehyde, then permeabilized with 0.1% Triton X-100 in PBS and blocked in 1% bovine serum albumin. For the immunofluorescent staining of cardiomyocyte-related proteins, cells were incubated in primary monoclonal antibodies to cardiac troponin T (cTnT), MEF2c and connexin43 (Cx43, all from Santa Cruz Biotechnology, SantaCruz, CA) at a dilution of 1∶100 for 120 min at room temperature, followed by TRITC-conjugated antibodies (Zhongshan Goldenbridge Biotechnology, Beijing, China) at a dilution of 1∶100 for 90 min at room temperature, and finally stained with DAPI (Sigma) for 5 min to label the nuclei at room temperature. For cytoskeletal staining, cells were incubated in Texas red isothiocyanate-conjugated phalloidin (Molecular Probes, Eugene, OR) for 30 min to stain all F-actin filaments and with DAPI for 5 min to label the nuclei at room temperature. All fluorescent images were taken under a Leica TCS NT confocal microscope (Wetzlar, Germany). In the experiments quantifying cTnT, cells were incubated in primary monoclonal antibodies to cTnT at a dilution of 1∶100 for 120 min at room temperature, followed by a horseradish-peroxidase (HRP)-conjugated antibody (Zhongshan Goldenbridge Biotechnology) at a dilution of 1∶100 for 90 min at room temperature, and finally stained with hematoxylin (Sigma) for 5 min to label the nuclei at room temperature. The signals were visualized using diaminobenzidine substrate (Sigma). The numbers of cultures were three totally. Under an Olympus IX71 microscope (Tokyo, Japan), three fields were randomly selected per culture and 100 cells were counted. Brown cells in color were taken as positive stained.

### Intracellular Ca^2+^ transient measurement

[Ca^2+^]i measurement using Fura-2 fluorescence was performed as previously described [3], [26].

### Atrial natriuretic peptide (ANP) quantification assays

Production of ANP released into the cell culture medium was measured by the enzyme linked immunosorbent assay (ELISA) using a Multiskan MK3 Ascent spectrophotometer as previously described [26].

### Western blotting

Total protein was extracted from rBMSCs using a standard method and quantified with a BCA protein assay kit (Pierce, Rockford, IL). Whole-cell protein extracts (20 µg/lane) were separated by SDS-PAGE and transferred to a polyvinylidene difluoride Immobilon-P membrane (Millipore, Bedford, MA) using an electroblotter (Bio-Rad). Membranes were blocked with Blotto B nonfat milk (Santa Cruz) for 30 min at room temperature, followed by overnight incubation at 4°C with primary antibodies to cTnT at a dilution of 1∶1000. Primary antibody binding was detected using a HRP-conjugated secondary antibody (Zhongshan Goldenbridge Biotechnology) and super ECL (Applygen, Beijing, China).

### Statistical analysis

Each experiment was conducted at least three times. All data were collected from cultures obtained from independent isolations. Statistical analysis was performed using one-way analysis of variance (ANOVA). A Student-Newman-Keuls (S-N-K) test was used to determine the difference between two groups within the multiple groups. In two group designed experiments, comparisons were done by using unpaired student's t-test. All data are expressed as mean±SD. Differences were considered significant when *P*<0.05. All the calculations were performed using SigmaPlot for Windows (version 11.0; Systat Software Inc., San Jose, CA).

## Results

### Effects of the magnitude of strain on the cardiac-related gene expressions of rBMSCs

**Figure 2:**
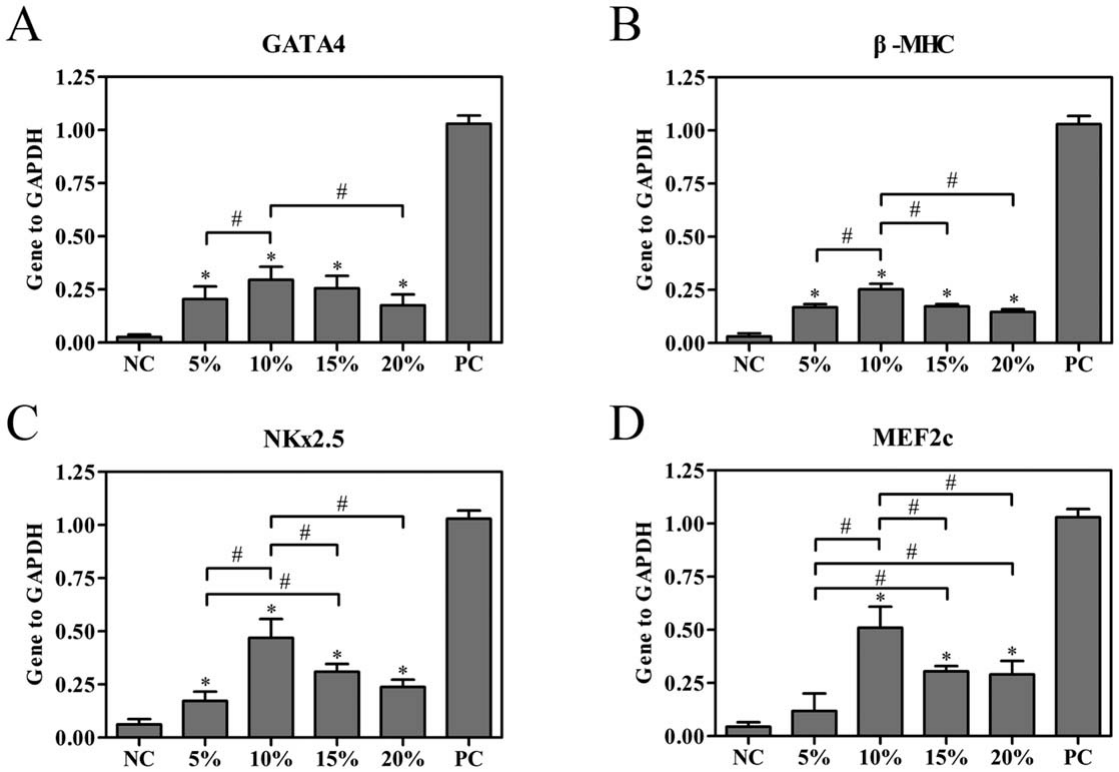
Effects of the magnitude of strain on the cardiac-related gene expressions of rBMSCs. rBMSCs grown on flexible membranes were subjected to 24 h of uniaxial strain resulting in 5%, 10%, 15%, and 20% elongation of the membrane at a frequency of 1 Hz and mRNA expression levels of GATA-4, β-MHC, NKx2.5 and MEF2c were evaluated on the 7^th^ day by quantitative real-time RT-PCR analysis. The expression of each gene was normalized based on the expression of GAPDH. (A) GATA4. (B) β-MHC. (C) NKx2.5. (D) MEF2c. NC, negative control (rBMSCs treated with regular complete medium); PC, positive control (normal neonatal rat cardiomyocytes). Results are shown as the mean±SD values (n = 4). *P<0.05 compared to the NC group; #*P*<0.05.

rBMSCs grown on flexible membranes were subjected to 24 h of uniaxial strain resulting in 5%, 10%, 15%, and 20% elongation of the membrane at a frequency of 1 Hz which were most commonly used in previous studies [29], [30] and then further cultured under normal culture conditions. Gene expression levels were determined on the 7^th^ day using quantitative real-time PCR analysis. As shown in Fig. 2, cyclic strain treatment induced the expression of GATA-4, β-MHC, NKx2.5 and MEF2c, an attribute that was absent in untreated cells (n = 4, *P*<0.05). Transcriptional expression of β-MHC, NKx2.5 and MEF2c increased in a strain-dependent manner for a magnitude of ≤10%. However, mRNA levels started to decrease when the strain magnitude reached 15%. Transcriptional expression of GATA-4 in 10% stretch group was higher than that in 5% stretch group (n = 4, *P*<0.05), and was higher than that in 20% stretch group (n = 4, *P*<0.05), but there was no significant difference between 10% stretch group and 15% stretch group (n = 4, *P*>0.05). According to MTT assay, there were no statistical differences detected among rBMSCs for different values of strain amplitudes (data not shown).

### Effects of the duration of strain on the cardiac-related gene expressions of rBMSCs

**Figure 3:**
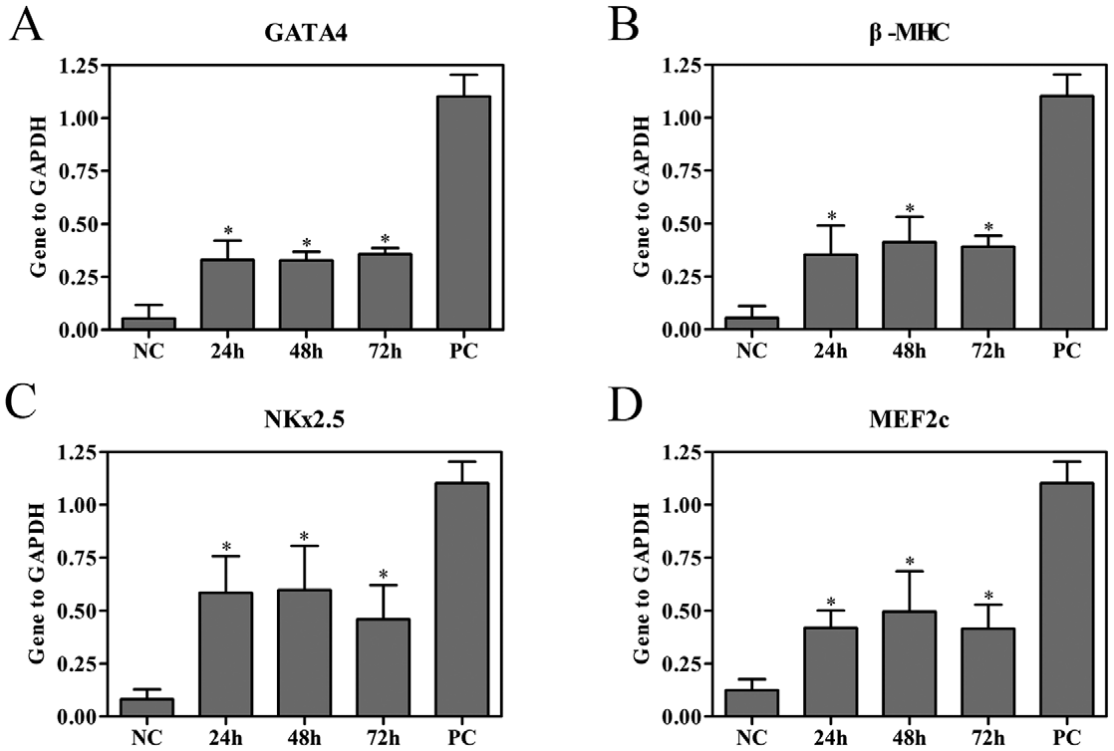
Effects of the duration of strain on the cardiac-related gene expressions of rBMSCs. rBMSCs were cultured under static conditions or exposed to cyclic strain of 10% at a frequency of 1 Hz for 24, 48 and 72 h and mRNA expression levels of GATA-4, β-MHC, NKx2.5 and MEF2c were assessed on the 7^th^ day by quantitative real-time RT-PCR analysis. The expression of each gene was normalized based on the expression of GAPDH. (A) GATA4. (B) β-MHC. (C) NKx2.5. (D) MEF2c. NC, negative control (rBMSCs treated with regular complete medium); PC, positive control (normal neonatal rat cardiomyocytes). Results are shown as the mean±SD values (n = 4). *P<0.05 compared to the NC group.

rBMSCs were cultured under static conditions or exposed to cyclic strain of 10% at a frequency of 1 Hz for 24, 48 and 72 h respectively, and mRNA expression levels of GATA-4, β-MHC, NKx2.5 and MEF2c were assessed on the 7^th^ day by quantitative real-time RT-PCR analysis. mRNA levels of GATA-4, β-MHC, NKx2.5 and MEF2c were significantly increased by strain at all time points compared with the static control cells (n = 4, *P*<0.05, Fig. 3). There were no significant differences in the expression of these cardiac-related genes among treatment times of 24, 48 and 72 h (n = 4, *P*>0.05, Fig. 3). According to MTT assay, there were no statistical differences detected among rBMSCs for different test duration (data not shown).

### Effects of the stretch frequency on the cardiac-related gene expressions of rBMSCs

**Figure 4:**
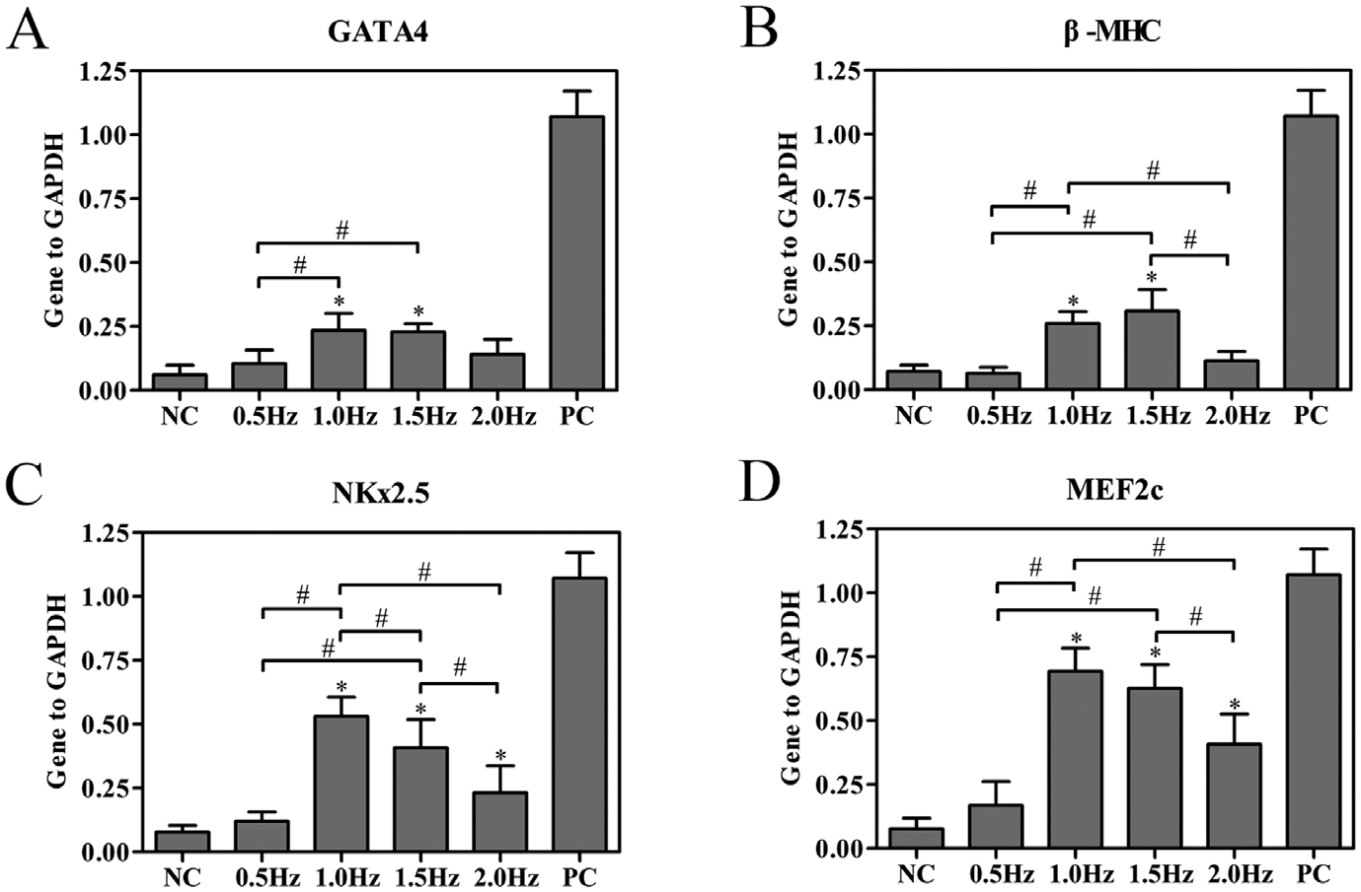
Effects of the stretch frequency on the cardiac-related gene expressions of rBMSCs. rBMSCs were cultured under static conditions or exposed to cyclic strain of 10% elongation at 0.5, 1, 1.5 and 2 Hz for 24 h and mRNA expression levels of GATA-4, β-MHC, NKx2.5 and MEF2c were assessed on the 7^th^ day by quantitative real-time RT-PCR analysis. The expression of each gene was normalized based on the expression of GAPDH. (A) GATA4. (B) β-MHC. (C) NKx2.5. (D) MEF2c. NC, negative control (rBMSCs treated with regular complete medium); PC, positive control (normal neonatal rat cardiomyocytes). Results are shown as the mean±SD values (n = 4). *P<0.05 compared to the NC group; #*P*<0.05.

rBMSCs were cultured under static conditions or exposed to cyclic strain of 10% elongation at 0.5, 1, 1.5 and 2 Hz respectively for 24 h, and mRNA expression levels of GATA-4, β-MHC, NKx2.5 and MEF2c were assessed on the 7^th^ day by quantitative real-time RT-PCR analysis. The expression levels of GATA-4 and β-MHC were significantly up-regulated at 1 Hz and 1.5 Hz (n = 4, *P*<0.05, Fig. 4A and B), whereas mechanical loading at 0.5 and 2 Hz had no evident effects on the expression levels of GATA-4 and β-MHC (n = 4, *P*>0.05, Fig. 4A and B). Analysis of NKx2.5 and MEF2c mRNA indicated increased expression at 1, 1.5 and 2 Hz of mechanical strain relative to unstretched cells (n = 4, *P*<0.05, Fig. 4C and D). The expression of NKx2.5 mRNA was significantly higher in the 1 Hz group than 1.5 Hz group (n = 4, *P*<0.05, Fig. 4C). According to MTT assay, there were no statistical differences detected among rBMSCs for different values of strain frequency (data not shown).

### Expression of cardiomyocyte-related proteins and response of calcium channels during rBMSC differentiation

**Figure 5:**
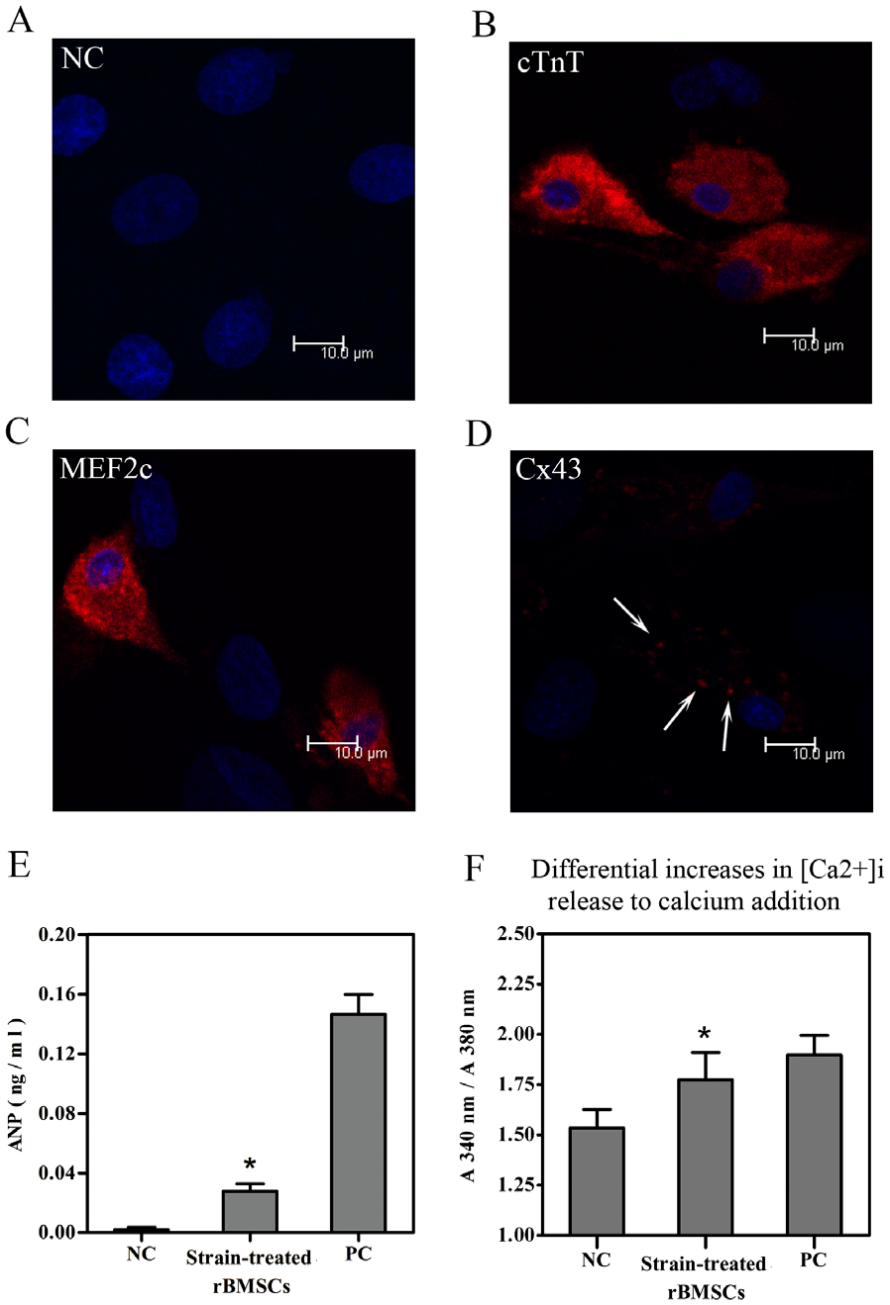
Expression of cardiac-related proteins and response of calcium channels during rBMSC differentiation. (A) Immunofluorescence staining of cTnT in the rBMSCs of the negative control group on the 14^th^ day. (B–D) Immunofluorescence staining of cardiomyocyte-related proteins in the rBMSCs on the 14^th^ day following 10% strain treatment at a frequency of 1 Hz for 24 h. Scale bar, 10 µm. (B) cTnT. (C) MEF-2c. (D) Cx43. (E) Secretion of ANP into the culture media assessed by ELISA. (F) [Ca^2+^]i level illustrated as the ratio between A_340 nm_ versus A_380 nm_ obtained upon calcium addition. NC, negative control (rBMSCs treated with regular complete medium); strain-treated rBMSCs (rBMSCs treated with 10% strain at a frequency of 1 Hz for 24 h and detected on the 14^th^ day); PC, positive control (normal neonatal rat cardiomyocytes). Results are shown as the mean±SD values (n = 3). *P<0.05 compared to the NC group.

For further analysis of cardiomyogenic differentiation induced by cyclic strain in rBMSCs, histological and immunohistochemical evaluations were performed. The effects of uniaxial strain on the expression of cardiomyocyte-related proteins were examined under test conditions of 24 h duration, strain frequency of 1 Hz and strain amplitude of 10%. The expression of cTnT, MEF-2c and Cx43 was evaluated using indirect immunofluorescence staining on the 14^th^ day. The expression of these cardiomyocyte-related proteins in the stretched rBMSCs was confirmed by confocal microscopy analysis (Fig. 5B–D). There was no staining for cTnT in the untreated cells (Fig. 5A). Moreover, the production of ANP which had been released into the cell culture medium was measured by ELISA. The analysis of culture supernatants showed a significant increase in the level of ANP protein after cyclic strain stimulation (Fig. 5E). In addition, to explore whether the cyclic strain-induced rBMSC-derived cardiomyocytes were functional, the difference in [Ca^2+^]i was calculated as the ratio between A340 nm versus A380 nm obtained upon calcium addition. The results revealed an enhanced response of cyclic strain-treated rBMSCs to calcium addition compared to the control (untreated cells) (Fig. 5F).

### Effects of cyclic strain or FSS on cardiomyogenic differentiation of rBMSCs

**Figure 6:**
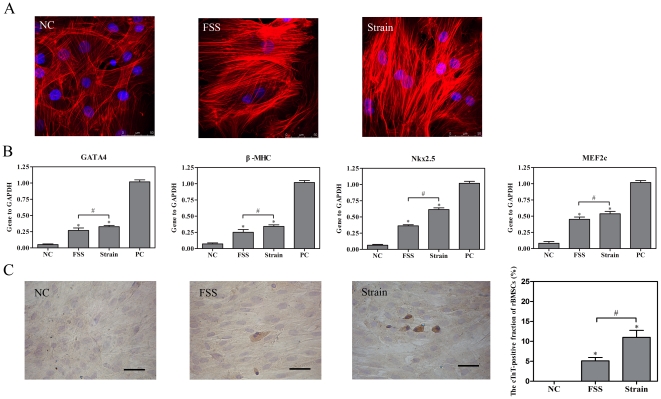
Effects of cyclic strain or FSS on cardiomyogenic differentiation of rBMSCs. rBMSCs cultured under static conditions, exposed to shear stress of 10 dyne/cm^2^ or exposed to cyclic strain of 10% at a frequency of 1 Hz for 24 h. (A) rBMSCs were incubated in Texas red isothiocyanate-conjugated phalloidin to stain all F-actin filaments (red) and with DAPI to label the nuclei (blue). Shear stress and strain were in left→right direction. Scale bar, 50 µm. (B) mRNA expression levels of GATA-4, β-MHC, NKx2.5 and MEF2c were assessed on the 7^th^ day by quantitative real-time RT-PCR analysis. (C) The expression of cTnT was assessed using indirect immunofluorescence staining on the 14^th^ day. Scale bar, 50 µm. NC, negative control (rBMSCs treated with regular complete medium); PC, positive control (normal neonatal rat cardiomyocytes). Results are shown as the mean±SD values (n = 3). *P<0.05 compared to the NC group; #*P*<0.05.

To compare cyclic strain and FSS on cardiomyogenic differentiation, rBMSCs were cultured under static conditions, exposed to shear stress of 10 dyne/cm^2^ or exposed to cyclic strain of 10% at a frequency of 1 Hz for 24 h. Confocal image of F-actin filaments showed that the cells cultured under static conditions had random fiber orientation. rBMSCs exposed to FSS were aligned parallel with the flow direction, whereas strained rBMSCs showed filaments aligned perpendicular to the axis of mechanical strain (Fig. 6A). Quantitative real-time RT-PCR analysis of cardiomyocyte-related markers on the 7^th^ day revealed that cells exposed to strain stimulation expressed GATA-4, β-MHC, NKx2.5 and MEF2c at a higher level than flow shear stimulation (n = 3, *P*<0.05, Fig. 6B). Immunocytochemical analysis of cTnT expression was assessed on the 14^th^ day. Compared with the shear stimulus, strain stimulus increased the proportion of cTnT-positive cells (n = 3, *P*<0.05, Fig. 6C). The results were in agreement with the real-time RT-PCR results.

### Biochemical and mechanical stimuli-induced rMSCs to differentiate into cardiomyocyte-like cells

**Figure 7:**
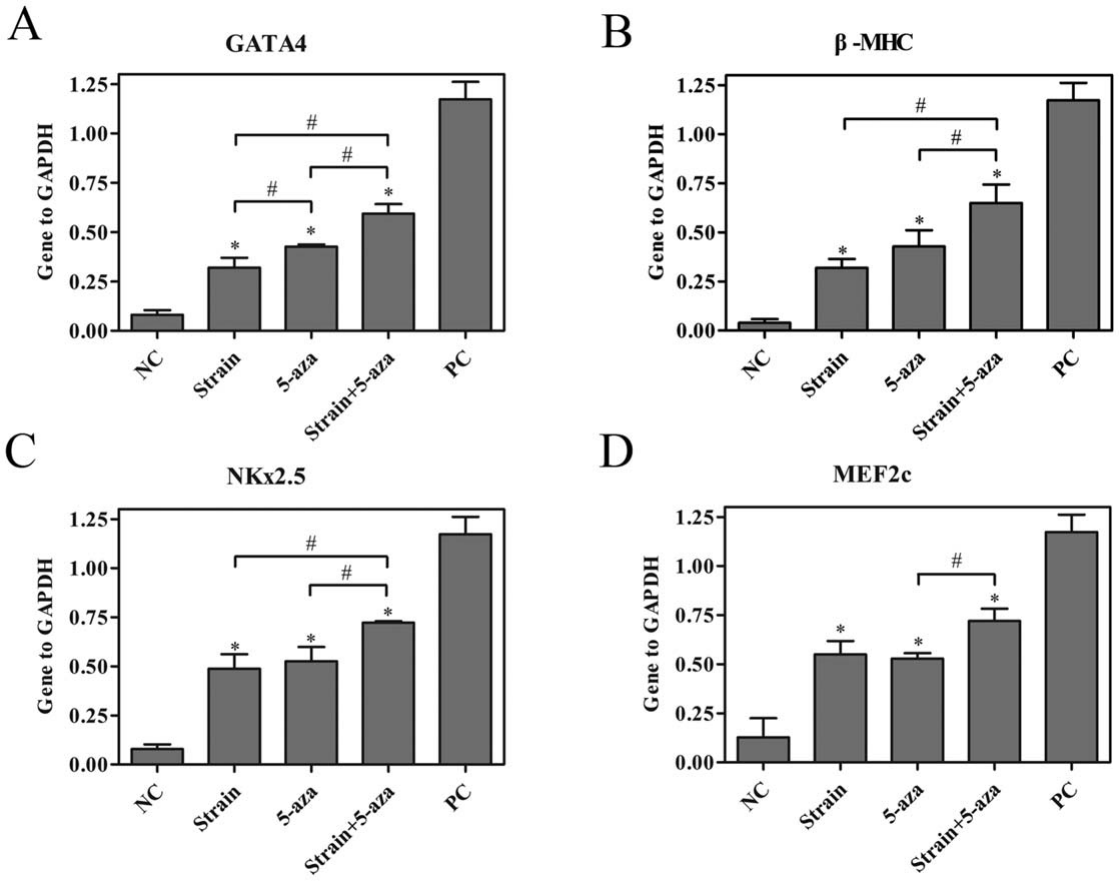
Effects of biochemical and physical stimuli on the cardiac-related gene expressions of rBMSCs. rBMSCs were cultured under static conditions, or exposed to cyclic strain of 10% at a frequency of 1 Hz, or given 10 µM 5-aza, or treated with cyclic strain of 10% at a frequency of 1 Hz combined with 10 µM 5-aza for 24 h. mRNA expression levels of GATA-4, β-MHC, NKx2.5 and MEF2c were assessed on the 7^th^ day by quantitative real-time RT-PCR analysis. The expression of each gene was normalized based on the expression of GAPDH. (A) GATA4. (B) β-MHC. (C) NKx2.5. (D) MEF2c. NC, negative control (rBMSCs treated with regular complete medium); PC, positive control (normal neonatal rat cardiomyocytes). Results are shown as the mean±SD values (n = 3). *P<0.05 compared to the NC group; #*P*<0.05.

To determine whether there was a synergistic or antagonistic effect of biochemical regents and mechanical physical force on cardiomyogenic differentiation, rBMSCs were cultured under static conditions, or exposed to cyclic strain of 10% at a frequency of 1 Hz, or given 10 µM 5-aza, or treated with cyclic strain of 10% at a frequency of 1 Hz combined with 10 µM 5-aza for 24 h. Quantitative real-time RT-PCR analysis of cardiomyocyte-related markers on the 7^th^ day revealed that a combination of cyclic strain and 5-aza had a stronger effect on mRNA expression of GATA-4, β-MHC, NKx2.5 and MEF2c than either treatment alone (n = 3, *P*<0.05), although the levels of these factors did not reach those for neonatal rat cardiomyocytes (Fig. 7).

**Figure 8:**
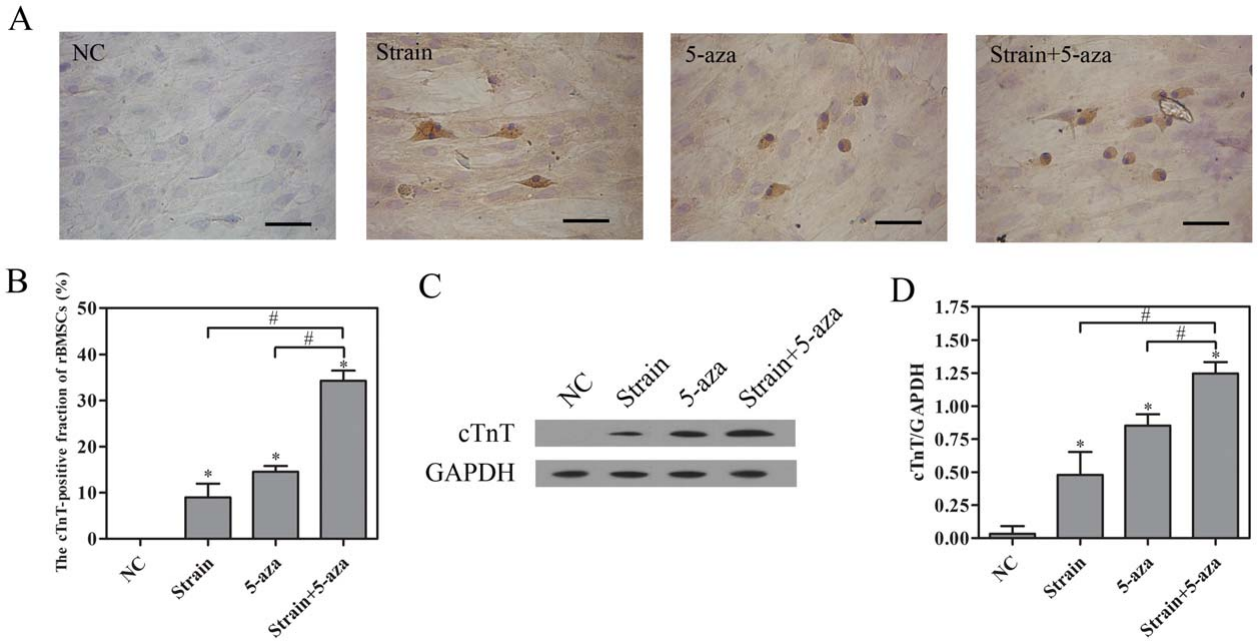
Effects of biochemical and physical stimuli on the expressions of cTnT in rBMSCs. rBMSCs were cultured under static conditions, or exposed to cyclic strain of 10% at a frequency of 1 Hz, or given 10 µM 5-aza, or treated with cyclic strain of 10% at a frequency of 1 Hz combined with 10 µM 5-aza for 24 h. The expression of cTnT was assessed on the 14^th^ day. (A) (B) Immunocytochemistry analysis of cTnT. Scale bar, 50 µm. (C) (D) Western blot analysis of cTnT. NC, negative control (rBMSCs treated with regular complete medium); PC, positive control (normal neonatal rat cardiomyocytes). Results are shown as the mean±SD values (n = 3). *P<0.05 compared to the NC group; #*P*<0.05.

To further investigate the effects of cyclic strain and/or 5-aza on rBMSC differentiation into cardiomyocyte-like cells, cTnT expression was assessed on the 14^th^ day by immunocytochemical analysis and western blotting. As shown in Fig. 8A, there was no cTnT-positive cell in the negative control group. A few cTnT-positive cells were detected after strain stimulation, and the number of which increased after 5-aza stimulation and combined with strain+5-aza stimulation. Combined strain+5-aza stimulation led to much stronger brown deposition compared to cyclic strain group, 5-aza group or the sum of the two groups (n = 3, *P*<0.05, Fig. 8A and B). As shown in Fig. 8C and D, compared to the negative control group, cTnT expression after strain and/or 5-aza stimulation increased especially after the combined strain+5-aza treatment. These results were identical with the RT-PCR results for mRNA levels of typical cardiomyocyte markers.

## Discussion

The majority of investigations involving cardiomyogenic differentiation of MSCs have focused on the effects of soluble stimuli [2]–[5], [8] or co-culture with cardiomyocytes [6], [7]. Our previous study has shown that rBMSC response to 10 dyne/cm^2^ FSS involves up-regulation of cardiomyocyte-related markers. The results of the present study revealed a higher increase in the cardiomyocyte-related markers at both mRNA and protein levels when rBMSCs were stimulated with cyclic strain than FSS. These changes in gene expression were related to the magnitude, duration and frequency of the cyclic strain. In addition, combined strain and 5-aza treatment yielded a stronger effect on cardiomyogenic differentiation of rBMSCs than either treatment alone.

The data of the present study conclusively demonstrate that mechanical strain induced cardiomyogenic differentiation of rBMSCs. Cardiac tissue is subjected to dynamic mechanical stresses from very early development, without a pause, during a person's entire life. Much of the heart experiences both active stretching during filling and self-generated mechanical force during ejection [32]. *In vitro* experiments revealed that static, dynamic stretch of neonatal or maturing cardiomyocytes could directly enhance the expression of several cardiac-specific genes [33], [34]. Another study showed that stretch induced cardiomyocyte hypertrophy and marked improvement of contractile function *in vitro*, which was closely resemble that of *in vivo* load-induced cardiac hypertrophy [29], [38]. The present study here confirmed that cyclic strain could induce transcriptional expression of the early cardiomyocyte-specific genes including GATA-4, β-MHC, NKx2.5 and MEF2c in rBMSCs, reflected by increased mRNA levels. However, the proliferation level of each group did not exhibit significant differences, so that the increase of gene expression was not due to cell proliferation, but the cyclic strain exertion. Differentiation is most often judged in terms of the upregulation of markers indicative of a mature, differentiated cell phenotype. These cardiomyocyte-specific genes play essential roles in early heart development by regulating expression of many genes that encode cardiac specific proteins [39]–[42]. This finding is consistent with previous studies about embryonic stem cells [29]–[31]. One such study showed that mechanical strain stimulated the mRNA expression of GATA-4 and MEF2c in mouse embryonic stem cells (ESCs) [29]. Furthermore, the expression of cardiomyocyte-related proteins including cTnT, MEF-2c, Cx43 and ANP substantially increased in the cells after induction by cyclic strain. Despite the up-regulation of the cardiac-specific genes and proteins, beating cells were not attained. It is possible that the cells were not mature enough or had not been cultured for an extended period of time, forming dense aggregates [30]. In addition, extracellular calcium leading to significant increase of calcium influx and intracellular calcium release demonstrated enhanced activity of L-type calcium channels. The calcium dependence of intracellular calcium release is based upon an increase in the trans-sarcolemmal flux of calcium [43]. These results indicate that the appropriate mechanical environment might be sufficient to induce stem cell differentiation into cardiomyocyte-like cells.

In this study, we investigated the influence of cyclic strain with different magnitude, duration and frequency on the cardiac-related gene expressions of rBMSCs. Our results showed that the cyclic strain at a frequency of 1 Hz or 1.5 Hz could induce rBMSC expression of the cardiac-related genes, and the cyclic strain at a frequency of 1 Hz was probably the best inducer, which might be related to the pace of heartbeat. It is interesting to note that the other studies have found that direct stretch of 10% strain with low frequency (10 cycles/min) could reduce differentiation of human embryonic stem cells, maintaining an undifferentiated state [32], [44]. In our study, cyclic longitudinal stretch of the cell culture surface was applied to rBMSCs in a controlled fashion using a stretch application device. The results demonstrated that strain amplitude of 10% at a frequency of 1 Hz for 24 h could be regarded as a proper abiotic elicitor of the cardiac-related gene expressions in rBMSCs. The 10% longitudinal stretch might be a physiologically relevant stimulus. In the whole heart, a 10% increase in cell length would result in a maximum of 1.331-fold increase in the end-diastolic volume. This magnitude of change in end-diastolic volume is well within that range observed in the intact heart [45], [46]. Koike et al. examined the effects of varying magnitudes of strain on the ST2 stromal cell line, showing that low levels of strain (1% and 5%) increased ALP activity and expression of Runx2. By contrast, high levels of strain decreased ALP activity (10% and 15% strain) [47]. Thus, for cardiomyogenic differentiation of BMSCs, 10% stretch might be the optimal stimulating load. Hamilton et al. demonstrated that long term cyclic strain (7 days) resulted in the expression of vascular smooth muscle α-actin and h1-calponin in rat bone marrow-derived progenitor cells [17]. The results of this study indicated that the 24 h treatment was adequate to the expressions of the cardiac-related genes in rBMSCs. Taken together, all of these results suggested that the effects of mechanical stimulus on MSCs should depend on the magnitude, duration and frequency of mechanical strain.

Results in the current study indicated that cyclic strain is a better stimulant of rBMSC differentiation toward the cardiomyocyte lineage than shear stress. During tissue development and remodeling, the cells are subjected to various forms of mechanical stimulation, which in turn shapes and regulates a large array of physiological and biological processes. In the present study, the stress fibers of rBMSCs orientated themselves parallel to the direction of flow and perpendicular to the direction of strain which are consistent with previous studies [17], [48]–[50]. This could be an adaptation process of the cells to minimize the amount of forces applied to cell bodies. The rearrangement of cytoskeleton caused by the mechanical signals is good for the extracellular signals to be transmitted into cells. Cells convert these mechanical signals into biochemical responses through a mechanism termed as mechano-transduction [51]. The precise mechanism that govern development of MSCs is still unknown, so future research is needed to decipher which signaling pathways are affected by mechanical loads. We also assessed the integrated effects of mechanical and biochemical stimuli on the cardiomyogenic differentiation of rBMSCs. Our results showed that combined application of strain and 5-aza to rBMSCs could result in a significant increase of the cardiomyocyte-related markers expression beyond that of either factor alone. The expression induced by 5-aza, consistent with data reported by other groups, may be associated with activation of the myogenic gene, MyoD, secondary to hypomethylation of selected cytosines involved in activating phenotype-specific genes [52], [53]. The mechanism for the synergistic effects of strain and 5-aza is likely to involve increased secretion of factors that accelerate cardiomyocyte growth and decrease potentially harmful effects of the nonspecific demethylating activity of 5-aza, but further investigation is required for confirmation.

MSCs have shown great promise in tissue repair. Before we can routinely use MSCs in human therapies, first we must understand thoroughly their responses to both mechanical and chemical factors [54]. The current study demonstrates that cyclic strain significantly induces cardiomyogenic differentiation of rBMSCs. Additionally, when rBMSCs were exposed to both cyclic strain and 5-aza, cardiomyogenic differentiation was significantly increased to a degree suggestive of a synergistic interaction. By combining different mechanical and cytokine modalities, one may be able to promote more rapid maturation of progenitor cell, thus improving graft stability more suitable for implantation. Furthermore, this work has far-reaching implications in terms of MSC differentiation under mechanical stimuli in bioreactors, and provides a basis for the design of new in vitro cell culture systems for stem cell and tissue engineering.
